# Dynamic Changes in Gut Microbiota Composition and Function over Time in Suckling Raccoon Dogs

**DOI:** 10.3390/ani16020188

**Published:** 2026-01-08

**Authors:** Shaochen Yu, Weixiao Nan, Zhipeng Li, Chongshan Yuan, Chao Xu

**Affiliations:** 1 College of Animal Science and Technology, Jilin Agricultural University, Changchun 130118, China; 2 Key Laboratory of Animal Production, Product Quality and Security, Ministry of Education, Jilin Agricultural University, Changchun 130118, China

**Keywords:** raccoon dog, gut microbiota, suckling period, metabolic function

## Abstract

Raccoon dog fur is a commercially valuable animal product. As the scale of raccoon dog breeding continues to expand, ensuring the health of these animals has become an urgent priority. The gut microbiota plays a central role in regulating animal health; however, current research on the composition of raccoon dog gut microbiota remains limited. This study aimed to characterize changes in the gut microbiota of suckling raccoon dogs across different stages, providing a foundation for future scientific feeding practices. Fecal samples of eight lactating raccoon dogs were collected and tested for microbiota on days 14, 21, and 45. Our results showed that the richness and diversity of microbiota increased with age in suckling raccoon dogs, peaking on the 45th day. Significant separation between groups was observed in both PCoA and NMDS analyses. UPGMA analysis indicated temporal fluctuations in gut microbiota composition. At the phylum level, Firmicutes and Bacteroidetes were the dominant taxa across all stages. LEfSe analysis at the genus level showed that *Bacteroides* was the most enriched taxon on the 14th day, *Fusobacterium* on the 21st day, and *Prevotella*_*9* on the 45th day. Tax4Fun and PICRUSt analyses identified metabolism and genetic information processing as the primary functional roles of the gut microbiota. Further investigation suggested that the microbiota may benefit raccoon dogs through membrane transport, carbohydrate metabolism, amino acid metabolism, and energy metabolism. These findings establish a theoretical basis for improving the survival rate of suckling raccoon dogs and developing scientifically informed feeding and management protocols.

## Simple Summary

Raccoon dogs, as important fur-bearing animals, generate multi-level employment opportunities across agricultural, industrial, and high-end fashion sectors. The survival rate of juvenile raccoon dogs directly determines the total number of animals available for pelt harvesting at year’s end, making scientific rearing of young animals crucial for enhancing economic returns. The gut microbiota plays a fundamental role in juvenile health, and the early postnatal period represents a critical window for microbial colonization. The initial establishment of microbial homeostasis not only influences immediate health status but also exerts long-term effects on metabolic, immune, and neurological development in raccoon dogs. However, the current understanding of gut microbiota dynamics in juvenile raccoon dogs remains limited. Therefore, this study investigated the temporal development of the gut microbiota in suckling raccoon dogs. This study showed that the richness and diversity of gut microbiota increased with age in suckling raccoon dogs. Firmicutes and Bacteroidetes are dominant phyla at each stage. Further research suggests that the microbiota may benefit raccoons through multiple metabolic pathways. These findings provide data support for improving the survival rate of suckling raccoon dogs.

## 1. Introduction

The raccoon dog (*Nyctereutes procyonoides*) is a carnivorous canid species native to East Asia, named for its morphological resemblance to raccoons (Procyonidae) [1]. But, there is no close genetic relationship between raccoon dogs and raccoons, on the contrary, raccoon dogs have a closer genetic relationship with canids such as domestic dogs [2]. During the early 19th century, the raccoon dog was introduced to the former Soviet Union for fur farming. Subsequently, the species was rapidly distributed across Western Europe, including Germany, the Netherlands, and Denmark [3]. As an introduced species, raccoon dogs have demonstrated remarkable ecological adaptability, enabling rapid population expansion and establishment as one of the most commercially important fur-bearing animals [4]. Raccoon dogs exhibit a defined suckling period from April to June annually, with weaning typically occurring at 45–60 days postpartum. The survival rate of suckling raccoon dogs are highly sensitive to multiple factors including genetics, nutrition, husbandry practices, and disease susceptibility [5]. Consequently, close monitoring of health parameters during this critical developmental stage is essential for effective management.

Host development and dietary habits are two primary determinants of gut microbiota composition [6]. Compared with herbivores, carnivorous animals typically exhibit reduced alpha diversity, whereas omnivores often demonstrate the highest beta diversity [7]. Herbivores possess complex digestive systems in which the gut microbiota plays an essential role in degrading recalcitrant substrates such as cellulose [8]. In contrast, carnivorous diets—which consist largely of proteins and lipids—are associated with lower microbial diversity [9]. Although the gut microbiota of raccoon dogs exerts a comparatively weaker influence on the host than in herbivores, it nonetheless contributes to nutrient metabolism [10], modulates growth [11], and plays a certain role in health maintenance and disease recovery [12,13]. The gut microbiota is altered by various factors such as diet, stress, host genetics, and physiological status [14,15]. Dysbiosis of the gut microbiota can contribute to various pathological conditions such as inflammatory disorders, metabolic dysfunction, and increased susceptibility to infections [16]. The gut microbiota and its host maintain a delicate symbiotic relationship, with gut microbial composition being intricately linked to host metabolic processes [10]. Conversely, the host’s health status critically influences gut microbiota stability [17]. These interactions suggest that microbial community shifts may serve as valuable biomarkers for assessing the physiological status of raccoon dogs. While age is a well-documented determinant of gut microbiota composition [18], current knowledge of raccoon dog gut microbiota remains limited, particularly during the suckling period. A comprehensive understanding of these microbial dynamics is essential for developing strategies to improve survival rates in suckling raccoon dogs.

In this study, we characterized the gut microbiota composition of raccoon dogs at postnatal days 14, 21, and 45 using 16S rDNA sequencing. Our objectives were to identify core microbial communities and elucidate their predominant metabolic pathways across different developmental stages. These findings provide a theoretical basis for optimizing raccoon dog breeding practices.

## 2. Materials and Methods

### 2.1. Sample Collection

The experimental animals were fed at the Zuojia Fox and Raccoon Dog Breeding Base in Jilin Province. All animal experiments were approved by the Ethics Committee of Jilin Agricultural University (No. 20230606001). In 14 days (D14), 21 days (D21), and 45 days (D45) after birth, eight litters of suckling raccoon dogs were randomly selected from each group, with one healthy raccoon dog selected from each litter. The main components of the diet for lactating female raccoon dogs were extruded corn, soybean meal, meat meal, fish meal, and blood meal. In the morning, raccoon dogs are in a relatively resting state and are prone to defecation. After a long rest at night, the intestinal environment is relatively stable and the microbiota is in a balanced state. Therefore, we chose to collect fecal samples in the morning. About 10 g of fresh fecal samples were collected during the morning period. The collected samples were stored in a liquid nitrogen tank using sterile gloves immediately after collection. Afterwards, the raccoon dogs whose feces were collected each time remained consistent. After all samples were adopted, we transferred them to the laboratory at −80 °C freezer until testing.

### 2.2. DNA Extraction and PCR Amplification

The DNeasy PowerSoil kit (QIAGEN, Hilden, Germany) was used to extract total DNA. The universal bacterial primers were 515F (CCTAYGGGRBGCASCAG) and 806R (GGACTACNNGGGTATCTAAT). Polymerase chain reaction (PCR) was used to amplify genomic DNA extracts. Universal bacterial primers were used to extract conserved bacterial 16S rRNA genes as described by Selvarajan [19]. PCR amplification was mixed 0.2 µmol/L primer, 15 µL PCR Master Mix, and 10 ng template DNA. The thermal cycling protocol was followed according to what has been previously reported [20]. The PCR thermal cycling protocol involved the sample being denatured 35 times at 98 °C for 10 s, followed by 30 s at 50 °C and 72 °C, followed by 5 min at 72 °C, and finally stored at 4 °C. The PCR products were purified using magnetic bead purification. After the concentration of PCR products was determined, 1% agarose gel was used to test the purity of PCR products. Variable regions 4 were amplified by PCR, and then detected by 2% agarose gel. Afterwards, the amplified products were purified, quantified, and combined before sequencing [21]. The library was sequenced on Illumina platforms after checked with Qubit (Thermo Fisher Scientific, Waltham, MA, USA).

### 2.3. 16S rRNA Sequencing Data Preprocessing

The data was separated based on the PCR primer sequence. The raw mate-paired fastq files were first subjected to quality filtering using the FastQC software (version 0.12.1) to obtain the optimized sequences. Flash (version 34.0.0.192) was used to concatenate the reads of each sample. Fastp (version 0.23.4) was used to filter and process the concatenated Raw Tags to obtain high-quality Tag data. Low-quality bases and data below 35 bp were trimmed to ensure a high-quality reading. To ensure the usability and accuracy of the dataset, Cutadapt (version 1.9.1) was used to remove primer sequences. The tags sequence was used to compare with the species annotation database to obtain the final valid data. The DADA2 algorithm within QIIME2 (version 2024.10) was employed to model and correct sequencing errors, inferring true amplicon sequence variants (ASVs). Subsequently, the Deblur plugin was applied for sequence denoising to obtain the initial ASVs set. Taxonomic classification of ASVs was performed using the Greengenes (https://ngdc.cncb.ac.cn/, accessed on 24 November 2025) reference database [22]. Following the generation of the ASV table, representative sequences, and taxonomic annotations, a phylogenetic tree was constructed, and analyses of species diversity and relative abundance were conducted.

### 2.4. Alpha Diversity Analysis and Beta Diversity Analysis

To evaluate the microbial richness and evenness, QIIME2 software (v2.0.6) was used to calculate Chao1, Dominance, Observed features, Pielou_e, Simpson, and Shannon. To compare differences between groups, PCA, PCoA, and NMDS were performed using the R-studio platform (version 4.3.2) [23]. The specaccum (version 2.7-2) function was used to generate species accumulation curves and display the upper and lower quartiles, median, and range of each sample point. Bray–Curtis, UniFrac, and Jaccard were used to calculate the distance matrix between pairs of samples. The tre function in QIIME2 (version 2024.10) was used to drawn the UPGMA clustering graph.

### 2.5. Significance Analysis of Intergroup Differences

LefSe (https://huttenhower.sph.harvard.edu/lefse/, accessed on 24 November 2025) was used to analyze the gut microbiota among different groups. The criteria for screening microorganisms were a LDA score greater than or equal to 4.0 and a *p*-value less than 0.05. LDA scores can reflect the magnitude of differences in abundance between groups, with larger absolute values indicating more critical species, while *p*-values can evaluate the statistical significance of differences. The Kruskal–Wallis rank sum test was used to screen for species with differences in component abundance. STAMP 2.1.3 version was also used as a graphical tool.

### 2.6. Functional Prediction

To assess the potential metabolic functions of gut microbiota, 16S rRNA sequence readings were clustered into ASVs, and the generated ASVs table was imported into PICRUSt [24]. The KEGG database (https://www.genome.jp/kegg/, accessed on 24 November 2025) was used to predict the functional content of microbial communities. The runRefBlast and make Function Prediction functions in R-studio (version 4.3.2) were used to analyze Tax4Fun [25,26]. After conducting closed reference clustering on the filtered sequence, BugBase website was used for functional prediction of data [27]. The FAPROTAX database (https://www.bic.ac.cn/, accessed on 24 November 2025) was used for functional annotation of cultivable microorganisms and prediction of microbial metabolic functions [28].

### 2.7. Statistical Analysis

The alpha diversity analysis was used for analysis of variance to calculate the significance between groups, and when *p* < 0.05, the difference was considered significant. All statistical analyses were conducted using GraphPad Prism version 10.0 (GraphPad Software, San Diego, CA, USA). Data are expressed as mean ± standard deviation (SD). Group differences were evaluated by one-way analysis of variance (ANOVA) followed by Tukey’s post hoc test, with a significance threshold set at *p* < 0.05.

## 3. Result

### 3.1. The Richness and Diversity of Gut Microbiota Among Young Raccoon Dogs of Different Ages

**Figure 1:**
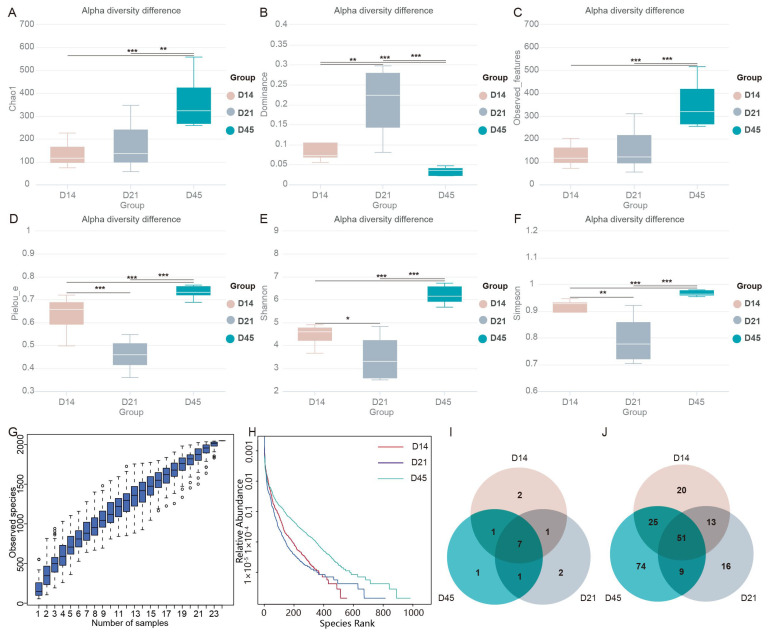
The richness and diversity of gut microbiota. (**A**) Chao1. (**B**) Dominance. (**C**) Observed_features. (**D**) Pielou_e. (**E**) Shannon. (**F**) Simpson. (**G**) Species accumulation box plot. (**H**) Rank abundance cure. (**I**) Phylum level Venn analysis. (**J**) Genus level Venn analysis. * *p* < 0.05, ** *p* < 0.01 and *** *p* < 0.001 indicate significant differences.

Alpha diversity can demonstrate species diversity within ecosystems (Figure 1A–F). The Chao1 estimator and observed_features in the D45 group was significantly higher than other groups, indicating that the species richness increased with age (Figure 1A). The Pielou_e index was used to measure the evenness of species. As shown in Figure 1D, the D45 group had the highest bacterial evenness. The dominance value of the D21 group was the highest, which further indicated that its bacterial evenness was reduced. Observed features, Shannon, and Simpson diversity indices were highest in the D45 group, indicating that community diversity increases with age (Figure 1E,F). With the increase in sample size, the position of the boxplot tends to flatten out, indicating sufficient sample size (Figure 1G). The Relative Abundance curve can be used to explain species abundance and species evenness, with a flatter curve indicating a more uniform distribution of species. The higher the abundance of species, the larger the range of the curve on the horizontal axis. As shown in Figure 1H, species abundance and evenness increase with age. Venn diagram analysis (Figure 1I,J) revealed that there were seven phylum-level microbial species shared among the three groups. In addition, there were two microbial species unique to group D14 and D21, respectively, and one bacteria unique to group D45. At the genus level, the D45 group had 74 unique microbial species, which was the highest among the three groups. Next was the D14 group, which had 20 unique species. The D21 group had the smallest number, with 16.

### 3.2. Differences in Gut Microbiota Among Young Raccoon Dogs of Different Ages

**Figure 2:**
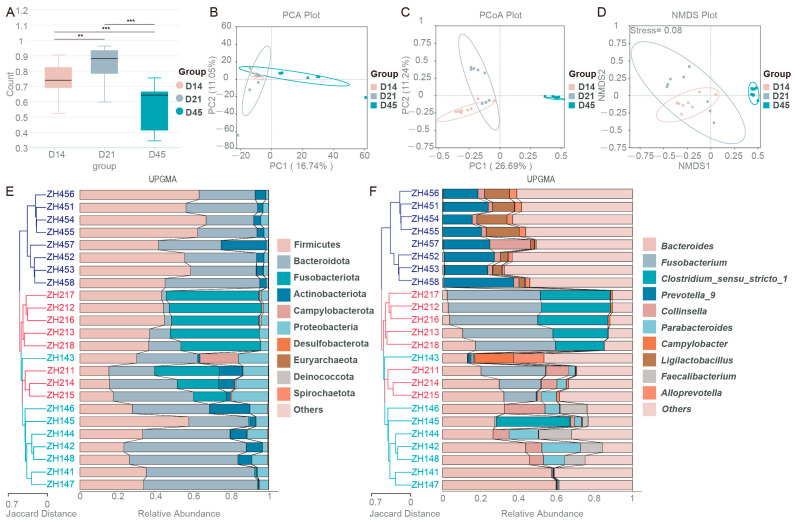
Differences in gut microbiota among different groups. (**A**) Beta index intergroup difference test. (**B**) PCA analysis. (**C**) PCoA analysis. (**D**) NMDS analysis. (**E**) UPGMA analysis at the phylum level. (**F**) UPGMA analysis at the genus level. ** *p* < 0.01 and *** *p* < 0.001 indicate significant differences.

In order to compare the difference between the groups, the Beta index inter group difference test was conducted. Figure 2A showed that the significant differences between the groups indicated that the age could affect the gut microbiota of raccoon dogs. PCoA showed significant differences in beta-diversity among the groups. PC1 and PC2 were 26.69% and 11.24%, respectively (Figure 2C). A clear separation of PCoA and NMDS was observed between the groups, indicating significant gut microbiota composition differences between different ages. The stress value of NMDS was 0.08, indicating that our results have reliable fitting (Figure 2D). The hierarchical clustering tree was used to show the structure of microbiota (Figure 2E,F). The result showed that D21 (ZH211–ZH218) and D14 (ZH141–ZH148) groups clustered together, while the D45 (ZH451–ZH458) group located on similar branches with the D21 (ZH211–ZH218) group.

### 3.3. Gut Microbial Community Composition

**Figure 3:**
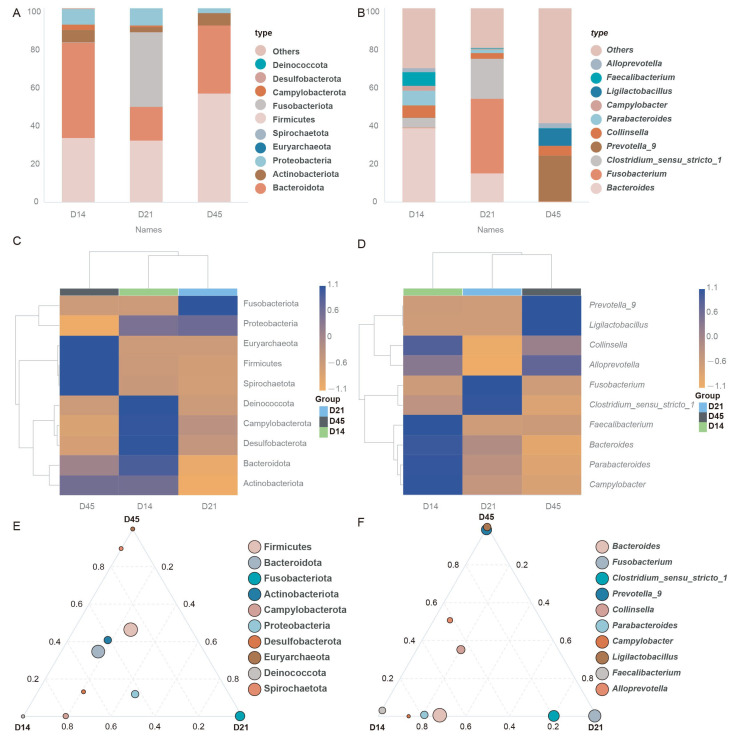
Gut microbiota community composition. (**A**) Column diagram of species at the phylum level. (**B**) Column diagram of species at the genus level. (**C**) Species abundance clustered heatmap at the phylum level. (**D**) Species abundance clustered heatmap at the genus level. (**E**) Ternary phase diagrams at the phylum level. (**F**) Ternary phase diagrams at the genus level.

The dominant phylum of group D14 were Firmicutes, Bacteroidota, Actinobacteriota, Proteobacteria, and Campylobacterota. In the D21 group, the predominant phyla were Firmicutes, Bacteroidota, Fusobacteriota, Actinobacteriota, and Protebacteria. The D45 group was enriched in Firmicutes, Bacteroidota, Actinobacteriota, and Protebacteria (Figure 3A). At the genus level, the gut microbiota of D14 was dominated by *Bacteroides*, *Clostridium*_*sensu*_*stricto*_*1*, *Collinsella*, *Feacalibacillus*, and *Parabacteroides*. In the D21 group, the predominant genera were *Bacteroidota*, *Fusobacterium*, *Clostridium*_*sensu*_*stricto*_*1*, *Collinsella*, and *Parabacteroides*. The D45 group was dominated by *Prevotella*_*9*, *Collinsella*, *Ligilactobacillus*, and *Alloprevotella* (Figure 3B). Compared with other groups, Deinococcota, Campylobacterota, and Desulfobacterota had higher abundance in the D14 group, Fusobacteriota and Proteobacteria had higher abundance in the D21 group, and Spirochaetota, Firmicutes, and Euryarchaeota had higher abundance in the D45 group at the phylum level (Figure 3C). At the genus level, *Faecalibacterium*, *Bacteroides*, *Parabteroides*, *Campylobacter*, and *Collinsella* had higher abundances in group D14, *Clostridium*-*sensu*_*stricito*_*1* and *Fusobacterium* had higher abundances in group D21, and *Prevotella*_*9*, *Ligulactobacillus*, and *AlloPrevotella* had higher abundances in group D45, compared with other groups (Figure 3D). In order to identify the differences in dominant species among the groups at different classification levels, including Phylum and Genus, we used ternary phase diagrams (Figure 3E,F) to demonstrate the different species in each group. The top 10 species with average abundance were selected to observe dominant species in different groups. Figure 3E showed that the dominant phylum-level species of Group D14 were Deinococcota and Campylobacterota, the dominant species of Group D21 was Fusobacteriota, and the dominant species of Group D45 were Euryarchaeota and Spirochaetota. At the genus level, *Feacalibacterium*, *Campylobacter*, *Parabacteroides*, and *Bacteroides* were dominant in group D14, *Fusobacterium* and *Clostridium*-*sensu*_*stricito*_*1* were dominant in group D21, and *Ligilactobacillus* and *Prebotella*_*9* were dominant in group D45 (Figure 3F).

### 3.4. Differential Abundance of Gut Microbiota Between Different Ages of Raccoon Dogs

**Figure 4:**
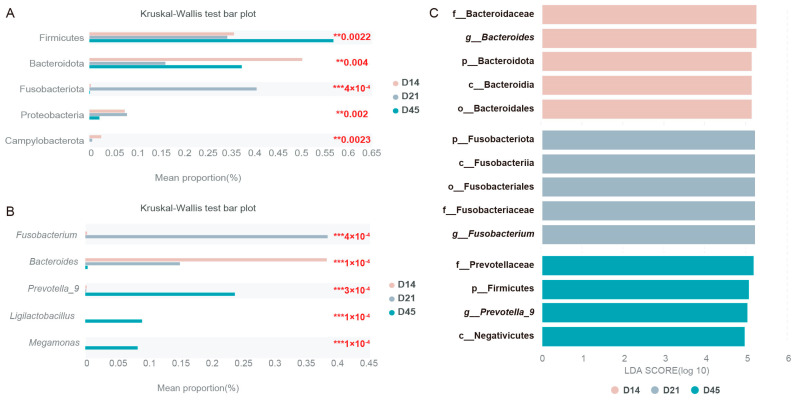
Differential abundance of gut microbiota between different groups. (**A**) Phylum level Kruskal–Wallis H-test bar plot. (**B**) Genus level Kruskal–Wallis H-test bar plot. (**C**) LEfSe analysis. ** *p* < 0.01, *** *p* < 0.001.

The differences in bacteria at the phylum and genus levels among different groups are shown in Figure 4A and Figure 4B, respectively. The taxonomic abundance of the gut microbiota of different groups were compared further using LEfSe analysis (LDA score > 4, *p* < 0.05). Bacteroidaceae, *Bacteroides*, Bacteroidota, Bacteroidia, and Bacteroidales were the most enriched taxa in the D14 group, while the enriched taxa of the D21 group were Fusobacteriota, Fusobacteriia, Fusobacteriales, Fusobacteriaceae, and *Fusobacterium*, while the D45 group had the least number of significantly enriched taxa, including Prevotellaceae, Firmicutes, *Prevotella_9*, and Negativicutes (Figure 4C).

### 3.5. Function Prediction of Gut Microbiota Between Different Ages of Raccoon Dogs

**Figure 5:**
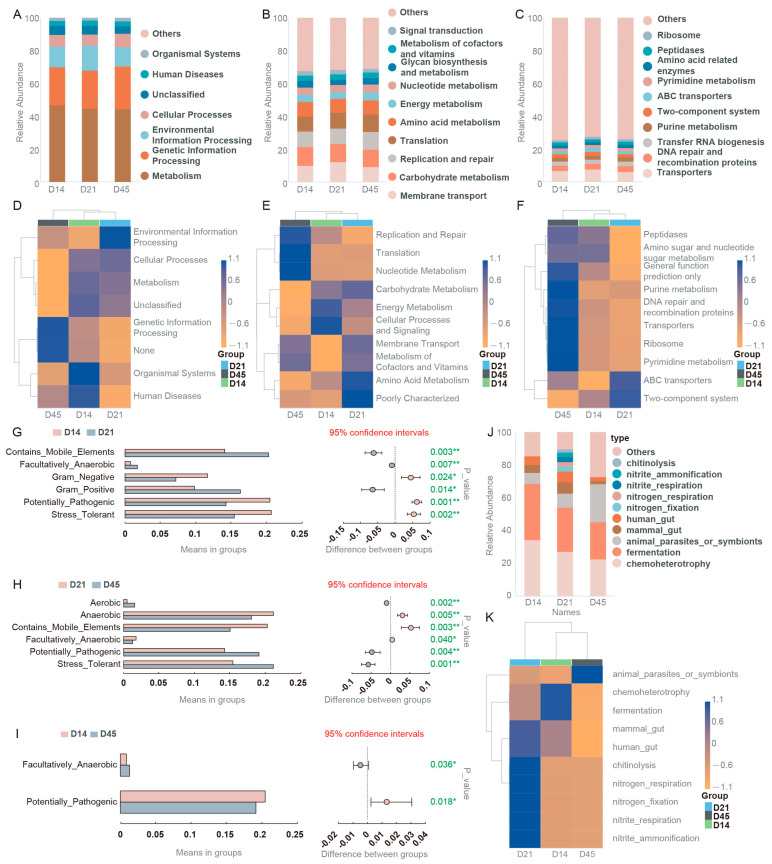
Function prediction of gut microbiota. (**A**–**C**) Tax4Fun analysis in different levels. (**D**–**F**) PICRUSt analysis in different levels. (**G**–**I**) BugBase analysis among the groups. (**J**,**K**) FAPROTAX analysis. * *p* < 0.05, ** *p* < 0.01.

In order to further explore the functions of gut microbiota among different groups, we used Tax4Fun to statistically analyze the top 10 functional information in terms of abundance at various annotation levels, and presented the results in a relative abundance bar graph. As shown in Figure 5A, the dominant functions in the level 1 were metabolism and genetic information processing. The main functions in the level 2 were membrane transport, carbohydrate metabolism, and replication and repair (Figure 5B). The main functions in the level 3 were transporters, DNA repair, and recombination proteins and transfer RNA biogenesis (Figure 5C). In addition, we obtained similar results through PICRUSt analysis (Figure 5D–F). The relative abundance of functions is shown in Figure 5G–I. Through BugBase analysis, we found that, compared with the D14 group, contains_mobile_elements, facultatively_anaerobic, and gram_positive were significantly up-regulated, while gram_negative, potentially_pathogenic, and stress_tolerant were significantly down-regulated in the D21 group (Figure 5G). Compared with the D14 group, facultatively_anaerobic was significantly increased, while potentially_pathogenic was significantly decreased in the D45 group (Figure 5H). Compared with the D21 group, aerobic, potentially_pathogenic, and stress_tolerant were significantly increased, anaerobic, contains_mobile_elements, and facultatively_anaerobic were significantly decreased in the D45 group (Figure 5I). Finally, we conducted cluster analysis on potential functions using FAPROTAX, as shown in Figure 5J, K, chemoheterotrophy and fermentation were significantly up-regulated in the D14 group. Chitinolysis, nitrogen_respiration, nitrogen_fixation, nitrite_respiration, and nitrite_ammonification were significantly up-regulated in D21 group. Animal_parasites_or_symbionts was significantly up-regulated in the D45 group.

## 4. Discussion

As commercially valuable fur animals, raccoon dogs have been extensively farmed worldwide. However, establishing standardized breeding protocols to enhance survival rates of suckling raccoon dogs remains a critical challenge in the industry. Gut microbiota homeostasis plays a pivotal role in regulating animal health, yet microbial composition is influenced by multiple factors including genetics, environment, diet, disease status, and particularly age. The current understanding of raccoon dog gut microbiota remains limited, with a notable knowledge gap regarding microbial dynamics during different suckling period. This study investigated compositional changes in gut microbiota across distinct suckling period in raccoon dogs.

Alpha diversity analysis revealed significantly higher Chao1, Shannon, and Simpson indices in 45-day-old raccoon dogs, demonstrating an age-dependent increase diversity and richness of gut microbiota. These findings align with previous observations in young children [29]. Relative abundance curves further corroborated this age-related progression in microbial community complexity. Notably, the Pielou_e index exhibited a transient decrease at day 21, while genus-level analysis identified greater species overlap between days 14 and 45, suggesting dynamic microbial succession during suckling period that warrants further investigation. Beta diversity analysis, including inter-group difference tests, PCoA, and NMDS, revealed significant compositional differences across developmental stages, confirming age as a determinant of microbial community structure [30]. UPGMA clustering demonstrated age-dependent grouping patterns, with temporally proximate samples showing greater microbial similarity. This dynamic microbial succession during suckling mirrors findings from prior studies [31].

The gut microbiota constitutes a highly complex ecosystem that maintains extensive interactions with the host. Its composition is shaped by multiple factors, including age, diet, and environmental conditions, with age representing one of the most influential determinants [32]. Disruption of gut microbial homeostasis is associated with gastrointestinal disorders, which can subsequently impair host growth performance [33]. In canids, gut microbiota composition exhibits limited variation at the phylum level. When summarizing the gut microbiota of dogs, it was found that Firmicutes, Fusobacteria, Bacteroidetes, Proteobacteria, and Actinobacteria are the main phyla at different age stages [34]. Similarly, as a member of the family Canidae, the gut microbiota of the blue fox is predominantly enriched with Firmicutes, Proteobacteria, Actinobacteria, Fusobacteria, and Bacteroidetes [35]. Analysis of gut microbiota composition in suckling raccoon dogs identified Firmicutes, Bacteroidota, Actinobacteriota, and Proteobacteria as the dominant phyla across all groups, consistent with previous findings [5]. Although the role of gut microbiota in carnivores may be limited compared to herbivores, the gut microbiome profiles of different species reflect their dietary composition [36]. Short-term consumption of a diet entirely composed of animal or plant products is sufficient to alter the microbial community structure [37]. Firmicutes were characterized by metabolic genes facilitating efficient energy harvest from dietary substrates and lipid storage in enterocytes [38], while Bacteroidetes mediated protein and carbohydrate degradation while serving as primary producers of short-chain fatty acids (SCFAs) that enhance intestinal barrier integrity through tight junction protein modulation [39]. Although less abundant, Actinobacteriota contributed significantly to microbial homeostasis, immune regulation, and metabolic health, with their depletion being associated with chronic disease states [40]. Proteobacteria, as early gut colonizers, exhibited ecological plasticity that makes their population dynamics a sensitive indicator of microbial dysbiosis [41]. Notably, we observed a marked increase in Fusobacteriota abundance at day 21, likely reflecting diet-induced microbial restructuring during the transition to solid-feed consumption [42].

Analysis at the genus level revealed distinct temporal patterns, with *Bacteroides*, *Fusobacterium*, and *Prevotella*_*9* showing significant abundance differences on days 14, 21, and 45, respectively. *Bacteroides*, a genus of anaerobic Gram-negative bacteria, possess recognized anti-inflammatory and immunomodulatory functions [43]. These bacteria are capable of converting dietary carbohydrates into host-absorbable short-chain fatty acids and secrete various proteases that support protein catabolism [44,45]. Additionally, through their involvement in bile acid metabolism, *Bacteroides* can influence microbial community composition and suppress pathogenic colonization [46]. In this study, *Bacteroides* emerged as a differentially abundant taxon during the early lactation phase in raccoon dogs, indicating its likely contribution to host developmental processes. The observed *Fusobacterium* surge on day 21 corresponds with the expected microbial shifts during weaning transition, as breastfeeding cessation initiates microbiota maturation toward adult-like compositions [47]. *Fusobacterium* is a Gram-negative anaerobic bacterium commonly found in the intestinal tract and oral cavity [48]. As a commensal organism, it contributes to host health by producing butyrate, which modulates immune responses and helps prevent pathogen colonization and invasion [49]. The abundance of *Fusobacterium* is often associated with dietary protein intake. In the present study, its abundance increased on day 14, likely reflecting elevated milk consumption during this period [50]. Previous studies have established that intestinal *Prevotella* abundance is generally correlated with diets high in carbohydrates, resistant starch, and fiber [51]. Consistent with this, fiber-rich nutritional interventions typically elevate *Prevotella* levels [52]. It is reported that adding Mannan oligosaccharides to the diet of raccoon dogs can alter the abundance of *Prevotella* in the gut, indicating that *Prevotella* is sensitive to dietary changes [5]. In the present study, we observed an increased abundance of *Prevotella-9* in the intestinal microbiota of the D45 group, which may be attributed to the introduction of extruded corn and soy-based protein in the diet during this stage. *Prevotella* is an important characteristic of the gut microbiota during the weaning stage, playing a crucial role in promoting growth performance and immune response [53]. This rise in *Prevotella-9* abundance further suggests the progression toward a more mature gut microbial community in suckling raccoon dogs [54].

The gut microbiota is now well-established to perform essential functions including polysaccharide digestion, vitamin/nutrient biosynthesis, and immune system regulation [55]. While 16S rDNA sequencing provides robust assessment of microbial composition, it cannot directly evaluate metabolic potential. To address this limitation, we employed PICRUSt and Tax4Fun for functional prediction. PICRUSt infers unknown gene content through an extended ancestral state reconstruction algorithm. Compared with the PICRUSt tool (version 2.6.0), Tax4Fun has a higher correlation between functional prediction and metagenomic mapping [56]. Functional annotation revealed metabolism and genetic information processing as the predominant pathways, consistent with the microbiota’s role in supporting host growth and development during suckling period. More specifically, we identified membrane transport, carbohydrate metabolism, and DNA replication/repair as key functional categories, demonstrating how gut microbes contribute to nutrient utilization and cellular maintenance in suckling raccoon dogs [57]. Our studies provide crucial data on the symbiotic relationship between gut microbiota and host development during this life stage. BugBase phenotypic prediction revealed significant variations in facultative anaerobes and potentially pathogenic bacteria across age groups, suggesting age-dependent shifts in microbial survival strategies and community assembly [58]. A thorough understanding of the early gut microbiota profile in raccoon dogs can offer critical insights into their health status during the suckling period. Targeted dietary modulation or direct intervention in the gut microbiota during early rearing may thus serve as a viable strategy to promote the growth and development of suckling raccoon dogs. While the functional regulation of host physiology by gut microbiota has gained increasing recognition, motivating extensive research into microbial composition and function, several limitations of this study warrant acknowledgment. Although 16S rDNA sequencing effectively profiled the gut microbiota, comprehensive functional characterization will require metagenomic approaches. Furthermore, the predicted microbial functions necessitate experimental validation through targeted metabolomic and proteomic analyses.

## 5. Conclusions

Although the gut microbiota of raccoon dogs has limited effects compared to herbivorous animals, revealing their microbiota characteristics is crucial for exploring the factors that affect the growth performance of suckling raccoon dogs. This study revealed dynamic temporal variations in the gut microbiota composition of suckling raccoon dogs, with significant structural differences observed across developmental stages. At the phylum level, Firmicutes and Bacteroidetes consistently dominated the microbial community throughout the suckling period. Genus-level analysis identified stage-specific enrichment patterns: *Bacteroides* predominated on day 14, *Fusobacterium* on day 21, and *Prevotella_9* on day 45. Functional prediction analysis suggests these microbial communities may contribute to host physiology through their involvement in carbohydrate, amino acid, and energy metabolism.

## Data Availability

All data and materials are available from the corresponding author upon request.

## References

[1] Lan T., Li H., Yang S., Shi M., Han L., Sahu S.K., Lu Y., Wang J., Zhou M., Liu H. (2022). The chromosome-scale genome of the raccoon dog: Insights into its evolutionary characteristics. iScience.

[2] Sugiura N., Tanaka A., Ochiai K., Yamamoto T., Morita T., Kato T., Kawamoto Y., Omi T., Hayama S.I. (2020). Association of sarcoptic mange with kinship and habitat use in raccoon dogs (*Nyctereutes procyonoides*). J. Vet. Med. Sci..

[3] Liang J., Wang T., Wang Q., Wang X., Fan X., Hu T., Leng X., Shi K., Li J., Gong Q. (2024). Prevalence of canine distemper in minks, foxes and raccoon dogs from 1983 to 2023 in Asia, North America, South America and Europe. Front. Vet. Sci..

[4] Minamikawa M., Ito M., Kovba A., Kobayashi Y., Abe G., Kooriyama T., Maeda K., Shimozuru M., Tsubota T., Sashika M. (2025). Epidemiological Survey of Canine Distemper Virus Infection: Exploring the Link Between Virus Spread and Invasive Raccoon (Procyon lotor) Population Growth in Hokkaido, Japan. Integr. Zool..

[5] Yuan C., Ren L., Sun R., Yun X., Zang X., Zhang A., Wu M. (2023). Mannan oligosaccharides improve the fur quality of raccoon dogs by regulating the gut microbiota. Front. Microbiol..

[6] Cheng C., Zhai H., Feng J., Zhang Z. (2025). Host Phylogeny and Feeding Habit Jointly Govern Mammalian Gut Microbiota Composition. Integr. Zool..

[7] Gharechahi J., Vahidi M.F., Sharifi G., Ariaeenejad S., Ding X.Z., Han J.L., Salekdeh G.H. (2023). Lignocellulose degradation by rumen bacterial communities: New insights from metagenome analyses. Environ. Res..

[8] Fujimori S. (2021). Humans have intestinal bacteria that degrade the plant cell walls in herbivores. World J. Gastroenterol..

[9] Ge Y., Zhu W., Chen L., Li D., Li Q., Jie H. (2021). The Maternal Milk Microbiome in Mammals of Different Types and Its Potential Role in the Neonatal Gut Microbiota Composition. Animals.

[10] D’Aquila P., Carelli L.L., De Rango F., Passarino G., Bellizzi D. (2020). Gut Microbiota as Important Mediator Between Diet and DNA Methylation and Histone Modifications in the Host. Nutrients.

[11] Panigrahi P. (2024). The neonatal gut microbiome and global health. Gut Microbes.

[12] Chaitman J., Gaschen F. (2021). Fecal Microbiota Transplantation in Dogs. Vet. Clin. N. Am. Small Anim. Pract..

[13] Ross F.C., Patangia D., Grimaud G., Lavelle A., Dempsey E.M., Ross R.P., Stanton C. (2024). The interplay between diet and the gut microbiome: Implications for health and disease. Nat. Rev. Microbiol..

[14] Sharma R.K., Azmi A., Kaka N., Sethi Y., Chopra H., Emran T.B. (2022). Role of gut hormones in diabetes mellitus: An update. Int. J. Surg..

[15] Ben Amor I., Chandran D., Amin R., Emran T.B. (2023). Nanotechnology’s advancement in diabetes mellitus regenerative medicine. Ann. Med. Surg..

[16] Fu Y., He Y., Xiang K., Zhao C., He Z., Qiu M., Hu X., Zhang N. (2022). The Role of Rumen Microbiota and Its Metabolites in Subacute Ruminal Acidosis (SARA)-Induced Inflammatory Diseases of Ruminants. Microorganisms.

[17] Qiu P., Ishimoto T., Fu L., Zhang J., Zhang Z., Liu Y. (2022). The Gut Microbiota in Inflammatory Bowel Disease. Front. Cell. Infect. Microbiol..

[18] Wang C., Deng W., Huang Z., Li C., Wei R., Zhu Y., Wu K., Li C., Deng L., Wei M. (2024). Nutrient Utilization and Gut Microbiota Composition in Giant Pandas of Different Age Groups. Animals.

[19] Selvarajan R., Sibanda T., Venkatachalam S., Ogola H.J.O., Christopher Obieze C., Msagati T.A. (2019). Distribution, Interaction and Functional Profiles of Epiphytic Bacterial Communities from the Rocky Intertidal Seaweeds, South Africa. Sci. Rep..

[20] Liu H., Xiao L., Liu Z., Deng Y., Zhu J., Yang C., Liu Q., Tian D., Cui X., Peng J. (2025). Impacts of Captive Domestication and Geographical Divergence on the Gut Microbiome of Endangered Forest Musk Deer. Animals.

[21] Zhu R., Fang Y., Li H., Liu Y., Wei J., Zhang S., Wang L., Fan R., Wang L., Li S. (2023). Psychobiotic Lactobacillus plantarum JYLP-326 relieves anxiety, depression, and insomnia symptoms in test anxious college via modulating the gut microbiota and its metabolism. Front. Immunol..

[22] Huang F., Li S., Chen W., Han Y., Yao Y., Yang L., Li Q., Xiao Q., Wei J., Liu Z. (2023). Postoperative Probiotics Administration Attenuates Gastrointestinal Complications and Gut Microbiota Dysbiosis Caused by Chemotherapy in Colorectal Cancer Patients. Nutrients.

[23] Calleros L., Barcellos M., Grecco S., Garzón J.P., Lozano J., Urioste V., Gastal G. (2024). Longitudinal study of the bovine cervico-vaginal bacterial microbiota throughout pregnancy using 16S ribosomal RNA gene sequences. Infect. Genet. Evol..

[24] Li W., Zeng X., Wang L., Yin L., Wang Q., Yang H. (2025). Comparative Analysis of Gut Microbiota Diversity Across Different Digestive Tract Sites in Ningxiang Pigs. Animals.

[25] Wemheuer F., Taylor J.A., Daniel R., Johnston E., Meinicke P., Thomas T., Wemheuer B. (2020). Tax4Fun2: Prediction of habitat-specific functional profiles and functional redundancy based on 16S rRNA gene sequences. Environ. Microbiome.

[26] Carter K.A., Fodor A.A., Balkus J.E., Zhang A., Serrano M.G., Buck G.A., Engel S.M., Wu M.C., Sun S. (2023). Vaginal Microbiome Metagenome Inference Accuracy: Differential Measurement Error according to Community Composition. mSystems.

[27] Bolyen E., Rideout J.R., Dillon M.R., Bokulich N.A., Abnet C.C., Al-Ghalith G.A., Alexander H., Alm E.J., Arumugam M., Asnicar F. (2019). Reproducible, interactive, scalable and extensible microbiome data science using QIIME 2. Nat. Biotechnol..

[28] Kuo J., Liu D., Lin C.H. (2023). Functional Prediction of Microbial Communities in Sediment Microbial Fuel Cells. Bioengineering.

[29] Roswall J., Olsson L.M., Kovatcheva-Datchary P., Nilsson S., Tremaroli V., Simon M.C., Kiilerich P., Akrami R., Krämer M., Uhlén M. (2021). Developmental trajectory of the healthy human gut microbiota during the first 5 years of life. Cell Host Microbe.

[30] Zhanbo Q., Jing Z., Shugao H., Yinhang W., Jian C., Xiang Y., Feimin Z., Jian L., Xinyue W., Wei W. (2024). Age and aging process alter the gut microbes. Aging.

[31] Gyriki D., Nikolaidis C.G., Bezirtzoglou E., Voidarou C., Stavropoulou E., Tsigalou C. (2025). The gut microbiota and aging: Interactions, implications, and interventions. Front. Aging.

[32] You I., Kim M.J. (2021). Comparison of Gut Microbiota of 96 Healthy Dogs by Individual Traits: Breed, Age, and Body Condition Score. Animals.

[33] Kim E.T., Lee S.J., Kim T.Y., Lee H.G., Atikur R.M., Gu B.H., Kim D.H., Park B.Y., Son J.K., Kim M.H. (2021). Dynamic Changes in Fecal Microbial Communities of Neonatal Dairy Calves by Aging and Diarrhea. Animals.

[34] Garrigues Q., Apper E., Chastant S., Mila H. (2022). Gut microbiota development in the growing dog: A dynamic process influenced by maternal, environmental and host factors. Front. Vet. Sci..

[35] Yuan C., Chen S., Sun R., Ren L., Zhao T., Wu M., Zhang A. (2024). Thymol improves the growth performance of blue foxes by regulating the gut microbiota. Front. Microbiol..

[36] Huang Z., Pan Z., Yang R., Bi Y., Xiong X. (2020). The canine gastrointestinal microbiota: Early studies and research frontiers. Gut Microbes.

[37] Pilla R., Suchodolski J.S. (2021). The Gut Microbiome of Dogs and Cats, and the Influence of Diet. Vet. Clin. N. Am. Small Anim. Pract..

[38] Zhang W., Ma C., Xie P., Zhu Q., Wang X., Yin Y., Kong X. (2019). Gut microbiota of newborn piglets with intrauterine growth restriction have lower diversity and different taxonomic abundances. J. Appl. Microbiol..

[39] Wan X.Z., Ai C., Chen Y.H., Gao X.X., Zhong R.T., Liu B., Chen X.H., Zhao C. (2020). Physicochemical Characterization of a Polysaccharide from Green Microalga Chlorella pyrenoidosa and Its Hypolipidemic Activity via Gut Microbiota Regulation in Rats. J. Agric. Food Chem..

[40] Tian B., Jiang Y., Liu R., Hamed Y.S., Rayan A.M., Xu S., Sun P., Yang K. (2024). Positive effects of extracellular polysaccharides from Paecilomyces hepiali on immune-enhancing properties by regulating gut microbiota in cyclophosphamide-induced mice. Int. J. Biol. Macromol..

[41] Moon C.D., Young W., Maclean P.H., Cookson A.L., Bermingham E.N. (2018). Metagenomic insights into the roles of Proteobacteria in the gastrointestinal microbiomes of healthy dogs and cats. Microbiologyopen.

[42] Lubin J.B., Silverman M.A., Planet P.J. (2024). Comparison of gnotobiotic communities reveals milk-adapted metabolic functions and unexpected amino acid metabolism by the pre-weaning microbiome. Gut Microbes.

[43] Zhang Y., Fan Q., Hou Y., Zhang X., Yin Z., Cai X., Wei W., Wang J., He D., Wang G. (2022). Bacteroides species differentially modulate depression-like behavior via gut-brain metabolic signaling. Brain Behav. Immun..

[44] Horvath T.D., Ihekweazu F.D., Haidacher S.J., Ruan W., Engevik K.A., Fultz R., Hoch K.M., Luna R.A., Oezguen N., Spinler J.K. (2022). Bacteroides ovatus colonization influences the abundance of intestinal short chain fatty acids and neurotransmitters. iScience.

[45] Mills R.H., Dulai P.S., Vázquez-Baeza Y., Sauceda C., Daniel N., Gerner R.R., Batachari L.E., Malfavon M., Zhu Q., Weldon K. (2022). Multi-omics analyses of the ulcerative colitis gut microbiome link Bacteroides vulgatus proteases with disease severity. Nat. Microbiol..

[46] Chen Z., Chen H., Huang W., Guo X., Yu L., Shan J., Deng X., Liu J., Li W., Shen W. (2024). Bacteroides fragilis alleviates necrotizing enterocolitis through restoring bile acid metabolism balance using bile salt hydrolase and inhibiting FXR-NLRP3 signaling pathway. Gut Microbes.

[47] Marrs T., Jo J.H., Perkin M.R., Rivett D.W., Witney A.A., Bruce K.D., Logan K., Craven J., Radulovic S., Versteeg S.A. (2021). Gut microbiota development during infancy: Impact of introducing allergenic foods. J. Allergy Clin. Immunol..

[48] John Kenneth M., Tsai H.C., Fang C.Y., Hussain B., Chiu Y.C., Hsu B.M. (2023). Diet-mediated gut microbial community modulation and signature metabolites as potential biomarkers for early diagnosis, prognosis, prevention and stage-specific treatment of colorectal cancer. J. Adv. Res..

[49] Wang X., Fang Y., Liang W., Wong C.C., Qin H., Gao Y., Liang M., Song L., Zhang Y., Fan M. (2024). Fusobacterium nucleatum facilitates anti-PD-1 therapy in microsatellite stable colorectal cancer. Cancer Cell.

[50] Robinson A.V., Vancuren S.J., Marcone M., Allen-Vercoe E. (2025). Characterization of diet-linked amino acid pool influence on Fusobacterium spp. growth and metabolism. mSphere.

[51] Tett A., Pasolli E., Masetti G., Ercolini D., Segata N. (2021). Prevotella diversity, niches and interactions with the human host. Nat. Rev. Microbiol..

[52] Ghosh T.S., Rampelli S., Jeffery I.B., Santoro A., Neto M., Capri M., Giampieri E., Jennings A., Candela M., Turroni S. (2020). Mediterranean diet intervention alters the gut microbiome in older people reducing frailty and improving health status: The NU-AGE 1-year dietary intervention across five European countries. Gut.

[53] Amat S., Lantz H., Munyaka P.M., Willing B.P. (2020). Prevotella in Pigs: The Positive and Negative Associations with Production and Health. Microorganisms.

[54] Zhao D., Liu H., Zhang H., Liu K., Zhang X., Liu Q., Wu Y., Zhang T., Zhang Q. (2022). Dietary supplementation with Cyberlindnera jadinii improved growth performance, serum biochemical Indices, antioxidant status, and intestinal health in growing raccoon dogs (*Nyctereutes procyonoides*). Front. Microbiol..

[55] Mo Z., Wang J., Meng X., Li A., Li Z., Que W., Wang T., Tarnue K.F., Ma X., Liu Y. (2023). The Dose-Response Effect of Fluoride Exposure on the Gut Microbiome and Its Functional Pathways in Rats. Metabolites.

[56] Sun S., Jones R.B., Fodor A.A. (2020). Inference-based accuracy of metagenome prediction tools varies across sample types and functional categories. Microbiome.

[57] Khan S., Chousalkar K.K. (2021). Functional enrichment of gut microbiome by early supplementation of Bacillus based probiotic in cage free hens: A field study. Anim. Microbiome.

[58] Li J., Chen Z., Yan X., Chen Q., Chen C., Liu H., Shen J. (2025). Effects of USP25 knockout on the gut microbial diversity and composition in mice. BMC Microbiol..

